# The Cleansing, Disinfection, and Protection of the Hands

**Published:** 1906-06

**Authors:** 


**The Cleansing, Disinfection, and Protection of the Hands.** By Dr. Carl S. Haegler. Translated by Charles Heron Watson, M.A., M.B. (Edin.). Pp. xix., 218. Edinburgh and London: William Green & Sons. 1906.

Until the present work was published, we believe there was but one book of importance in the English language which dealt in a thorough manner with the subject of the disinfection of the hands, though in the German language there are many. For this reason, as well as for the intrinsic merits of Haegler's researches and reflections on the subject, we cordially welcome the appearance of his book in an English garb.

The practical surgeon, not himself a skilled bacteriologist, may perhaps feel a disposition to regard the investigation of the problems of hand-disinfection as a simple matter. The perusal of such a book as that under consideration will, in such case, show him that the truth is far otherwise. The investigations, to be at all reliable, have to be carried out with the most scrupulous care and attention to detail. False conclusions, based upon faulty methods of procedure and an imperfect realisation of the possible sources of error, are as easy to obtain as reliable conclusions are difficult. In the present work are summarised the results obtained after twelve years of patient labour and thought by a skilled surgeon and bacteriologist, and an account of the methods which were employed in the investigations. Most of the questions which daily confront the practical surgeon when preparing the hands for an operation have been made the subject of study, such as the localisation of micro-organisms in the hands; the efficiency of the various methods devised for the removal of these organisms, as, for example, the efficiency of the different modes of mechanically cleansing the hands and of cleansing them with such disinfectants as spirit soap, antiseptic soap, corrosive sublimate lotion, and alcohol; the degree of bacteriological cleanliness it is possible to obtain at the beginning of an operation and to maintain during its course; the value of operating gloves, &c. Lastly prophylaxis, the prevention of infection and the general care of the hands, is considered.

The translation appears to us to have been excellently made; we forget as we read that the book is a translation, for the translation has been into English, not into German-English.

